# Content validity of the EQ-HWB and EQ-HWB-S in a sample of Italian patients, informal caregivers and members of the general public

**DOI:** 10.1186/s41687-024-00706-y

**Published:** 2024-03-23

**Authors:** Sara Masutti, Camilla Falivena, Fredrick Dermawan Purba, Claudio Jommi, Clara Mukuria, Aureliano Paolo Finch

**Affiliations:** 1 Simon-Kucher and Partners, Milan, Italy; 2 Centre for Research on Health and Social Care Management (CERGAS), SDA Bocconi School of Management, Bocconi University, Milan, Italy; 3 Faculty of Psychology, Universitas Padjadjaran, Jatinangor, Indonesia; 4 Department of Pharmaceutical Sciences, University of Eastern Piedmont, Novara, Italy; 5 Sheffiled Centre of Health and Related Research, University of Sheffield, Sheffield, UK; 6 EuroQol Office, EuroQol Research Foundation, Rotterdam, The Netherlands; 7 Health Values Research and Consultancy, Amsterdam, The Netherlands

## Abstract

**Background:**

The EuroQol Group recently developed two new instruments, the EQ Health and Wellbeing (EQ-HWB) and the EQ Health and Wellbeing short version (EQ-HWB-S). The EQ-HWB and EQ-HWB-S are intended to capture a broad range of health and broader quality of life aspects, which may be relevant to general public members, patients, their families, social care users and informal carers. This study assesses the content validity of the Italian version of the two instruments in a sample of Italian patients, social care users and informal carers.

**Methods:**

Participants were recruited using a convenience sampling approach. One-on-one interviews were carried out using video-conferencing interviews. A semi-structured topic guide was used to guide the interview procedures, with open-ended questions supplemented by probes. Participants were asked to explain important aspects of their health and quality of life, to complete the questionnaires and verbalize their thoughts.

**Results:**

Twenty participants comprising of patients (n = 9), informal carers (n = 6), and members of the general public (n = 5) participated to the study. Content validity was summarized into six main themes: comprehension, interpretation, acceptability, relevance, response options and recall period. All participants found the instruments easy or quite easy to understand and to respond to. Items were relevant for all three groups of participants, and response options appropriate.

**Conclusions:**

The Italian version of the EQ-HWB showed content validity in measuring health and wellbeing in a mixed Italian population.

## Background

Economic evaluations are commonly used in the health technology assessment of treatments and interventions. Their main outcome measure is quality-adjusted life years (QALYs), which encompasses in a single metric survival (i.e., length of life) and health-related quality of life (HRQoL) [1]. QALYs are anchored to a 1 to 0 scale, where 1 corresponds to full health and 0 to death. There are different methods to put the “Q” into QALYs i.e., measuring and valuing HRQoL, and these include the use of vignettes (i.e., scenarios describing the impact of a condition), eliciting values directly from patient populations and using generic preference-based measures (GPBM) of health [2, 3].

There are numerous GPBMs in the literature, including the most commonly used EQ-5D [4, 5], Short-Form 6 dimensions (SF-6D) [6], and Health Utilities Index Mark 3 (HUI3) [7], the Quality of Wellbeing Self-Administered Scale (QWB-SA) and the Assessment of Quality of Life 8 dimensions [8, 9]. The validity and responsiveness of these GPBMs have been assessed in multiple health conditions, disease areas, cultural contexts, and numerous populations, showing in many cases results in support of the instruments [10]. Nevertheless, GPBMs were developed for measuring differences in health, and in that they may include dimensions and items that are too narrow for detecting important differences in other contexts where broader QoL aspects may matter [11]. For example, GPBMs have been criticized for not capturing aspects relevant to informal carers, social care users, and those with long-term conditions [12].

Other measures have been developed specifically targeting aspects relevant for other users. For example, the Caregiver Burden Interview was developed for caregivers, while the Adult Social Care Outcomes Toolkit (ASCOT) includes aspects important to social care users [11, 13, 14]. Yet, when conducting economic evaluations, using different measures in combination, such as a GPBM of health and a social care measure, may be difficult for different reasons. Using different outcome measures makes it difficult to make comparisons across sectors. Combining outcomes from different measures may not be possible but even where they can be combined, this may result in double counting of similar aspects covered by different instruments. A solution that has been proposed is to develop a single instrument that covers aspects important to users and beneficiaries of health and social care services.

The EuroQol Group recently developed two new instruments aimed at cross-sector comparisons, the EQ Health and Wellbeing (EQ-HWB) and EQ-HWB short version (EQ-HWB-S). The EQ-HWB (25 items) and EQ-HWB-S (9 items) cover items related to seven dimensions: activity, relationships, cognition, self-identity, autonomy, feelings and physical sensations. In doing this, they capture a range of health and broader quality of life (QoL) aspects, which may be relevant for use among members of the general public, patients, their families, social care users and informal carers. The EQ-HWB-S classifier is amenable to valuation, and therefore is intended for use in economic evaluation across health, social care and public health sectors. The measures were developed based on domains identified in a large qualitative review on the QoL aspects that patients, social care users and informal carers identified as important followed by item generation and both qualitative and quantitative testing of the selected items in these populations [15, 16]. Development and testing of items was undertaken in English (source version) and three other languages: Simplified Chinese, German and Argentinian Spanish. Both the EQ-HWB and EQ-HWB-S are experimental instruments, meaning that their descriptive system needs further testing and may still be modified.

Available guidelines such as the COnsensus-based Standards for the selection of health Measurement Instruments (COSMIN) advocate for testing measures in all languages and populations for which instrument usage is intended [17]. One aspect of testing that is important for instruments is assessing the validity of the measure, that is, whether the instrument measures what is says it measures. Content validity is one type of validity testing that focuses on “the degree to which the content of an instrument is an adequate reflection of the construct to be measured” [18]. It includes assessment of face validity in any new language version, which addresses the extent to which an instrument appears to measure what it claims to measure, usually with non-experts (e.g., not instrument developers or clinicians). COSMIN guidance highlights the importance of assessing content validity as it gauges how well an instrument encompasses all relevant aspects of the construct it aims to measure, and it aids researchers in determining the measurement efficacy of research instruments. While the EQ-HWB and EQ-HWB-S development studies investigated the content validity of individual items to inform their selection in different languages and in mixed populations representing the target populations [19], there is limited evidence on the performance of the two newly developed instruments as a whole. Moreover, no study has investigated the content validity of the Italian versions of the instruments. The current study assesses the content validity of the Italian EQ-HWB and EQ-HWB-S in a sample of the Italian population composed of patients, informal carers, and healthy individuals.

## Methods

The focus of this study was on the face validity of the measures, i.e., how comprehensive, comprehensible and relevant the items of the Italian version of the EQ-HWB and EQ-HWB-S are in a target population of potential users to ensure that the measures and the translation were valid. Qualitative interviews using cognitive debriefing were carried out between July and September 2020. Interviews aimed to explore potential users’ viewpoint on the items of the EQ-HWB and EQ-HWB-S and the overall questionnaires, and understand how different groups (i.e., patients, informal carers, general population) interpreted them. A translation from English (Appendix Table 6) to Italian was performed, based on international translation guidelines [19]. The translation included two parallel forward and backward translations, reconciliation of translated versions, approval from external independent reviewer and approval of final translated version from the study team.

### Recruitment and consent

Participants were recruited using convenience sampling using work/wider acquaintances and volunteer contacts through some local patient associations. A quota was set on whether participants were members of the general public (25–30%), patients (40–45%) and carers (25–30%) as these groups were the target users of the measure. No quota was set for the sample in terms of gender, age or education, albeit a balance in these characteristics was considered whenever possible. COSMIN guidance suggest that instruments are assessed for content validity with a minimum of 7 participants [20] as this is considered sufficient to gain an understanding of the meanings attributed by the target population to the instruments. Participants were contacted via email and provided information regarding the study using an information sheet and a consent form that was signed before the interview.

### Procedures

Cognitive debriefing was used to test any difficulty in understanding and answering the Italian version of the questionnaire [21]. Cognitive debriefing uses both think-aloud and retrospective probing methods. Think-aloud implies oral verbalization of the thought process of participants. Retrospective probing aims at asking participants further questions based on their feedback.

Interviews were conducted in Italian by a single interviewer (SM) who had previous qualitative interview experience. Interviews were carried out one-on-one with each participant via a video-conferencing service (i.e., Zoom). The selection of this administration method was based on the safety constraints imposed by the COVID-19 pandemic in Italy. Additionally, its feasibility for interviews conducted in the Italian context had been demonstrated by previous studies [22].

A semi-structured topic guide was developed (Appendix Table 5). The guide comprised open-ended questions and tasks, which were supplemented with retrospective probing, where necessary. The interview procedure consisted of 6 parts. First, an explanation of the scope and outline of the interview was given to participants and they were given the chance to ask questions. Then, a familiarization session on the process of thinking aloud was carried out where participants were asked to verbalize their thoughts while thinking about the place where they lived in. Participants were then asked to reflect on what health and quality of life meant to them in order to identify what was important to participants and how this related to the questionnaires. Finally, the think-aloud exercise started. The draft Italian EQ-HWB experimental version (Appendix Table 6), preceding the 1.0 version, was shown on the screen and the interviewees were asked to complete each item while verbalizing their thoughts. Probes were used to further investigate aspects of the interview related to the face validity, including difficulties in understanding the questions and if there were any irrelevant or redundant questions. Participants were then asked to complete a ranking exercise of the five most important items, to identify if the items of the short version of the questionnaire, the EQ-HWB-S, matched participants’ viewpoint. The EQ-HWB interview preceded the EQ-HWB-S one where participants were just asked to read the questionnaire and comment aloud (see Appendix Table 5).

### Data analysis

Interviews were audio recorded and transcribed verbatim in Italian before the analysis. Transcribed interviews were analysed independently by two members of the team (SM, CF) using thematic analysis using a framework approach [23]. An existing coding framework on content validation of different preference-based measures [24] was adapted and used in this study, as shown in Appendix Table 7. This includes face validity aspects regarding what participants think of items including comprehension (e.g., the use of odd or difficult wording that was unfamiliar), interpretation (e.g., difficulties with interpretation or wrong, too narrow or too wide interpretations), response options, acceptability and relevance of the items. This approach was considered appropriate as there were pre-existing questions related to face validity that were being addressed (e.g., comprehension). Transcripts were reviewed and a list of key topics and macro-themes was developed. Transcripts were indexed and sections were exported in Microsoft Excel. Analyses were supplemented by coding in Italian language using Nvivo (i.e., transcripts have been double-coded by each independent coder). A matrix with all themes and participants was created and comments in each cell were refined by moving sentences to the macro-theme of reference. Information that was not relevant to face validity was not included in the analysis. An interpretation of the results followed. The translation of the analysis to English happened for write-up and discussion.

Qualitative research can be influenced by researchers’ experience and ideological background. The use of an existing framework helped to minimize the impact of the personal influence of the researchers on the team. Independent double-coding by researchers who were not involved in the development of the measure ensured that they were not influenced by the views of the developers. Results were subsequently reviewed—still independently—by the rest of the team including by a researcher who was involved in the development of the source English measure (CM) which was important in the context of interpreting the results in light of what the instruments were designed to measure. The findings were then jointly discussed at the end of the process. The audit trail was documented to ensure transparency and traceability throughout the research.

## Results

### Participants

A total of 35 participants were invited to take part in the study, 20 of whom agreed. The majority of participants were patients (n = 9, 45%), followed by informal carers (n = 6, 30%) and members of the general public (n = 5, 25%). Demographic information of the participants is provided in Table 1. Among patients, 3 had an oncological disease, 3 a rheumatic disease, and 3 another disease i.e., cardiovascular, inflammatory and degenerative disorders. Participants who reported caring for others were female, and were providing informal care to patients affected by oncological (n = 3, 50%), neurodegenerative (n = 2, 35%) or inflammatory (n = 1, 15%) disorders. Some participants reported having “good” or “very good” knowledge of health and other questionnaires (n = 11, 55%), while others (n = 9, 45%) reported having “theoretical”, “scarce” or “no” knowledge of them.

**Table 1:** Socio-demographic characteristics of the sample

Variable	Group	**N = 20** (%)
Gender	Female	14 (70%)
	Male	6 (30%)
Age (years old)	25–34	6 (30%)
	35–44	4 (20%)
	45–54	6 (30%)
	55+	4 (20%)
Profession	Employee or autonomous worker	12 (60%)
	Retired	3 (15%)
	Student	4 (20%)
	Unemployed	1 (5%)
Civil status	Married or cohabiting	13 (65%)
	Single	7 (35%)
Education	Any university degree	15 (75%)
	High school diploma	4 (20%)
	Middle school licence	1 (5%)
Medical education	No	16 (80%)
	Yes	4 (20%)
Participant category	General public	5 (25%)
	Carers	6 (30%)
	Patients	9 (45%)
Level of experience with health and/or social care questionnaires (e.g., knowledge of EQ-5D or a similar questionnaire)	None	6 (30%)
	Scarce	2 (10%)
	Theoretical	1 (5%)
	Good	9 (45%)
	Very good	2 (10%)

The following key is used to identify participants in this paper: GP refers to the general public, P indicates patients, C represents carers, M stands for male, F denotes female, and numbers are used to indicate the sequential order of the participants during interviews.

### Meaning of health and quality of life

Most participants found it difficult to draw marked distinctions between the concepts of health and QoL. Discussions of what health and QoL meant resulted in a number of themes, some of which are more aligned with the meaning of health traditionally reported in the literature, while most of them are aligned with the definition of QoL. The identified themes related to the presence or absence of diseases, the ability to perform activities, social participation, emotional functioning, living conditions and the possibility of accessing services.

Some patients interpreted the meaning of QoL as strictly related to health, and in light of having a disease or highlighted the differences between having a disease or being in good health. For example, one patient (CF5) argued that QoL is “when your health interferes with your daily life. Even an occasional malaise, or a recurrent one, can affect your daily life and alter your wellbeing”. Another patient (PF3) argued that QoL “means the “maximum”: having a good life, being physically, morally and psychologically healthy” and a general public participant (GPM1) agreed by stating that “to understand QoL you need to look at physical wellbeing, mental [wellbeing], let’s say the 360 degrees wellbeing of an individual”. Other participants considered QoL as not overlapping with health. One carer (CF4) stated that “in reality […] my experience makes me think that the concept of QoL, of a life that has quality, and it is worth living, can coexist with having health conditions and precarious health”.

Health was often considered as the possibility of performing activities. For example, one participant (PF1) argued “for me [health] is doing the little daily things, a walk, shopping, work activities” and another that “it is even simply knowing what you can do in one day, how many things you can do”.

Participation was mentioned as an important aspect of someone’s QoL. For example, participants mentioned that QoL are “the level […] of your community participation, the level of your relationships, […] aspects such as your family” and “the social interaction with other people” (PF1).

Different nuances of the emotional functioning theme were touched upon by participants. In particular, health was also associated with “spiritual” wellbeing (PM2), “peacefulness” (GPF2) and “personal satisfaction” (PF5). QoL was associated with mental wellbeing, such as feeling “tranquil” (PF5).

Another theme identified by participants was living conditions, which included autonomy, not relying on others’ help and having access to basic needs. For example, one carer (CF6) explained that QoL is being independent, as it is shown if you are “able to take care of yourself without help and support of others”, while a member of the general public (GPF5) stated that it is “having good food, a clean environment, being able to exercise”.

The possibility of accessing services was mentioned by some participants. For example, a carer (CF6) mentioned that QoL was “living […] with the possibility of accessing services […] without big difficulties”, and a carer (CF1) mentioned that QoL is “having assistance, the minimum level of assistance of an individual” (Table 2).

**Table 2:** Sub-themes related to the meaning of health and QoL, and frequency of responses

Themes	Sub-themes	Frequency of response
Presence or absence of health	Disease consequences	8
	Mental and physical wellbeing	7
	Physical wellbeing	6
	Mental wellbeing	6
Activities and participation	Social relationships	5
	Work	4
	Day-to-day activities	3
	Hobbies, passions, free time	1
Living conditions	Autonomy	3
	How your life impact others	1
	Routine	1
Access to services	Social assistance	3
	Presence of infrastructures	1
Emotional functioning	Emotions	1
	Satisfaction and achievement	1
	Positive feelings	1
	Spirituality	1

### Face validity

Face validity results are available in Table 3. Broadly, participants found the EQ-HWB easy or quite easy to understand, and they considered the items included relevant and comprehensive. No important aspects of QoL was identified as missing and just one participant found the EQ-HWB too long. Some participants suggested clarifications in the wording of some items, which are reported below.

**Table 3:** Response issues identified for the EQ-HWB self-administered experimental version, preceding 1.0 version

^**^Relevance issues identified for the EQ-HWB-S self-administered experimental version, preceding 1.0 version. With regards to comprehension, recall, interpretation, response option selection and acceptability, these are considered as overlapping for the two questionnaires

#### Comprehension and interpretation

All participants stated that the questionnaires were “easy” or “quite easy” to complete. Although participants identified no odd wording (i.e., with unusual terms or sentences), items related to control (item 6), hopelessness (item 13) and feeling accepted by others (item 19) needed further explanation or an example to aid understanding.

Participants could generally easily interpret the EQ-HWB and EQ-HWB-S and their items, although sometimes (i.e., 15 times) they faced difficulties in the interpretation of the items. Items 4 (ability to do day-to-day activities), 6 (control over day-to-day life), 13 (hopelessness) and 19 (acceptance) were difficult for more than one participant, while items 16, 17, 18, 21, 24/25 were only difficult for one participant each.

For four participants (GPM1, GPF2, PF1, PM2), difficulties emerged from the fact that items could be equally interpreted as related to physical, or emotional aspects. This was related to items 4 (ability to do day-to-day activities), 6 (control over day-to-day life), 13 (hopelessness), 17 (sleep issues) and 18 (feeling exhausted). For example, in relation to items 4 (ability to do day-to-day activities), one participant (GPF2) argued that “within the questionnaire, I will think about physical difficulties. If you asked me this question outside of the questionnaire, my answer would have been related to concentration or time management”. For item 6 (control over day-to-day life), the same participant could not discriminate whether the question was related to physical or psychological aspects, even though this item had an example providing further information. In relation to the item 18 on feeling exhausted, one participant (GPM1) argued that “exhausted can mean being unmotivated, […] sad, […] or on the other hand […] being physically tired”. The participant argued this ambiguity could result in inconsistencies between participants.

Two participants (GPF2, PM2) found the item 6 on control over day-to-day activities difficult to interpret, as the word “control” is not frequently used in the Italian language in this context and may be understood as quite a harsh term to indicate the ability to manage or influence the events and circumstances in one’s life.

Three participants (PM2, CF5, CF6) found the item 13 on hopelessness difficult to interpret, in the Italian version (Italian translation: *“Ho sentito di non avere aspettative”).* This was because, as one participant (CF6) argued, “[it is unclear if we] are talking about distrust, pessimism […] or expectations from other people. Or is it simply not having goals, not having a purpose?”. A proposed solution was to add an example so that the item could be better defined.

As for item 19 on feeling accepted by others, two participants (GPM3, GPF4) from the general public noted that the item could have some difficulties in the interpretation because of different reasons. The first participant (GPM3) argued that it is difficult to understand the item from the perspective of a participant with no/few social contacts. The second participant (GPF4) argued that because the example in the item referenced “feeling like you are able to be yourself”, the item could also be interpreted as self-acceptance.

For one participant (PF1), the examples given in specific questions may have added to the difficulty in interpreting the questions. For example, for item 4 on the ability to do day-to-day activities, she considered that this item could be answered in different ways because “there are different types of work, housework is [for example] more [physically] strenuous [than other works]”. The same participant (PF1) reported that item 16 on feeling unsafe could be answered in different ways as it covered different issues, as “fear of falling” could be frequent in older adults, whereas “abuse or other physical harm” should never happen. Both these questions have examples or explanations which are aimed at aiding comprehension.

Additional information in items can also come from combining aspects in a single question, for example, sad and depressed. Two participants (PF8, GPF5) reported problems in relation to this double-barrelled item “sad” and “depressed”, as they argued that these are very different feelings, and that while sadness is familiar and normal to most, depression is a disease.

#### Acceptability: inappropriate/judgemental questions

Generally, the instruments were considered acceptable and appropriate. Four participants (PF1, PM2, PF4, PF8), who were all patients, reported issues with the acceptability of some items. Issues of acceptability were related to what may be considered extreme ends of the scale i.e. very bad or too good. Two patients (PF1, PM2) found the item 13 on hopelessness associated with stigma, as having “nothing to look forward to” was perceived as an emotionally burdensome topic for them to think about. For example, one participant (PM2) was interpreting it as “a moment of resignation, apathy” and asked if there could be a better way to formulate it, likely due to the profound emotions and strong impact it might have on respondents. On the other hand, one participant (PF4) felt that the item 20 on feeling good about yourself is inappropriate for patients as, in case of sickness, nobody feels good. Moreover, one participant (PF8) felt that the term “unable” in item 7 on inability to cope with day-to-day life was correct but a bit heavy to read for patients.

#### Relevance

No participants suggested additional domains for the EQ-HWB. Six participants found that there were close similarities between some items, which meant one or the other could be removed. More specifically, two participants (GPF2, GPF4) identified overlaps between item 4 on the ability to do day-to-day activities or item 6 on having control and item 7 on coping with day-to-day life.

Similarities between item 20 on acceptance by others and item 19 on feeling good about themselves were also noticed by a patient (PF1), because “in my opinion, if you feel good about yourself, you can also be in balance with the others”.

Irrelevant items were identified three times only, regarding item 11 (feeling frustrated), 13 (hopelessness) and 23 (severity of physical pain). Participants identified other items that were more relevant, namely item 12 on feeling sad/depressed (instead of items 11 and 13) and 22 (frequency of physical pain). In no cases more than one participant agreed on the irrelevance of an item.

#### Response options

Most participants felt that the response options were appropriate and easy to select. Five participants (GPM1, GPF2, PM2, GPM3, PM7) found difficult to distinguish between “only occasionally” and “sometimes” for the item control over day-to-day life and for the item feeling good about themselves. One participant (GPM3) felt that feeling accepted by others had too many response options, albeit no other participant reported problems regarding this item.

One participant (PF8) found that the term “unable” (translated in original language with the word “inabile”) was not appropriate, as it could be potentially considered judgmental in Italian. In fact, “unable” is often used as a derogatory term in common language, and used as a synonym of “incompetent, inadequate, sloppy”.

#### Recall period

The use of a 7-day recall period was generally considered appropriate by the current study participants. One participant (GPF4) stated that use of this recall period is easier for day-to-day and other types of activities than for emotions (i.e. such as the feeling of loneliness). In fact, this participant noticed that when the item does not begin with “I felt”, it seems a “more real and objective fact […], against a situation where a person has to remember the perception of that feeling of danger or loneliness”.

### Ranking

Table 4 reports the result of the ranking exercise. Overall, the items considered most relevant were ability to do day-to-day activities, receiving support by people, control over own life, performing basic care needs, autonomy, independence, ability to get around indoors/outdoors) and ability to do activities.

**Table 4:** Ranking of the most relevant questions for the different groups of participants: General public, patients, carers

* where nothing is written, no participants included the item in their top-5

Legend for the % of participants including the item in their top-5:

*Note* The order of the items reflects an early version of the questionnaire. **Items in bold**: included in the EQ-HWB-S. Questions in bold are EQ-HWB-S. EQ-HWB-S indicates short version of EQ Health and Wellbeing.^a^*EuroQol Research Foundation. EQ-HWB*^*TM*^ *is a trade mark of the EuroQol Research Foundation*

There were differences in the support given to the items by different groups of participants. More specifically, items related to support, assistance, control over day-to-day activities and mobility were more frequently identified as relevant by patients and carers than by members of the general public. In contrast, loneliness and being accepted by others were ranked more frequently as important by members of the general public.

For carers, the most frequently endorsed items were day-to-day activities, coping with life and feeling unsupported by people. For patients, the most frequently endorsed items were day-to-day life, mobility, control over one’s life and feeling unsupported.

## Discussion

This study evaluated the content validity of the Italian version of EQ-HWB and EQ-HWB-S by assessing their face validity within an Italian cohort, encompassing patients, social care users, and informal carers. The findings offer valuable insights into the usability of the instrument and highlight significant areas of concern that need attention for optimal utilization—some which relate to the translation and some to the face validity of items. Moreover, this study builds upon existing evidence supporting the efficacy of the instruments, as reported from various perspectives in previous research [15, 16, 25–33].

The study found evidence in support of the face validity of the Italian EQ-HWB and EQ-HWB-S. The instrument was relevant for different participants in a cohort of Italian subjects, including patients, carers and members of the general public. Both the EQ-HWB and EQ-HWB-S were easy to understand and to respond to. No participant identified missing aspects of health or QoL, showing the instrument is comprehensive. Minor issues were also identified, for example in the interpretation of the items or because of the presence of similar response options and items (each of these minor issues was identified more than 5 times by participants, whereas all other response issues were identified less than 5 times). The EQ-HWB-S version had items that were less likely to be endorsed as being problematic.

The way participants thought about health and QoL was along themes of physical and mental health, and absence of disease. Other themes were also related to broader constructs, such as social relations. These themes are similar to those identified in other studies, for example they are consistent with the evidence collected by Penton et al. [24], that also found that patients/members of the public interpreted health and QoL in terms of physical, mental health and other constructs such as social relations or ability to perform daily activities. They also closely reflect the WHO definition of health, which encompasses “complete physical, mental and social wellbeing” [34]. The relevance of these themes supports the core domains of the EQ-HWB and EQ-HWB-S and provide further suggestions for development and adaptability to a local context.

Some participants mentioned in their definition of health and QoL, that part of it is the possibility of accessing services that are needed. Such interpretation may be influenced by the current state of play in Italy, where accessibility to healthcare services (especially specialized care) is increasingly a problem, due to accessibility barriers, such long waiting times [35].

The ranking exercise identified the importance of the items on being able to do daily activities, autonomy and independence, and support from others. These results are aligned with previous studies [24, 36, 37], that also found physical functioning, autonomy and relationship with others as a notable aspect in the conceptualization of health and QoL. In addition, the items that were considered the most important by the sample are included in the short instrument, the EQ-HWB-S, apart from item 15 on support from others. In fact, this item was considered relevant by 44% (n = 4) of patients and 50% (n = 3) of carers in the ranking exercise, but was not included in the short questionnaire. Moreover, item 18 on feeling exhausted, that was not selected in the ranking exercise by the participants, was included in the EQ-HWB-S. This outcome confirms the selection of the most relevant items for the EQ-HWB-S in an Italian context, but also suggests some potential differences. Among carers and patients, themes like support and/or assistance, control over day-to-day activities and mobility were identified as relevant in the ranking exercise more frequently than the general public although the socio-demographic background and the relative familiarity of the sample with health and/or social care questionnaires might have also played a role. A recent study assessing different measures in the context of cancer found that the EQ-HWB-S was considered to be relevant but no generic measure covered all the concepts identified as important in patients with cancer [38]. The difference across the groups highlights the challenges of developing generic measures that are applicable across different populations including cultural contexts.

A relevant result that emerged was that some participants (i.e., 5) found it difficult to distinguish the response options “sometimes” and “only occasionally”. In Australian face validity study of older people (n = 24) using an English version, one person considered these options to be the same [39]. Face validation and psychometric assessment of the items in the source version and the three other languages in the development study did not highlight problems with individuals completing the items [16, 26]. The English version of the EQ-HWB-S has been valued in a feasibility study with evidence that individuals can distinguish between these two levels although the difference in disutility is not large e.g. −0.031 and −0.034 for “only occasionally” and “sometimes” being sad/depressed [40]. Consideration of alternative distinct response levels in the Italian and other language versions may be warranted which is important for valuation.

Participants provided suggestions related to the wording and the translation of the items. The translatability of concepts in a different language, Italian in this case, may indirectly have affected interpretation of some items. For example, item 13 on hopelessness posed challenges to be interpreted in the Italian version (Italian translation: “Ho sentito di non avere aspettative”). To address this, a suggested solution was to include an example to provide a clearer definition. Participants highlighted the significance of incorporating examples to guide responses and offer a more comprehensive context for interpretation, a point that was brought up several times during the discussions. Some other interpretation issues emerged, mainly in relation to topics that could have touched upon both physical and psychological aspects, leaving participants unable to correctly interpret the item by themselves. There is a potential risk that these issues impact responses and the validity of comparisons across participants, leading to biased scores and decision making. These results are aligned with those provided by the study conducted in Argentina [28], Australia [39] and USA [38] in terms of issues of interpretation of some items for some individuals. Some issues with the acceptability were identified by participants in the group of patients. It was suggested to soften them by replacing the term “unable” or have “nothing to look forward to”, as they are associated with stigma or they might not be accepted by some categories of participants. Identification and testing of a more appropriate translations and/or alternative wording may improve the instruments although this can be challenging for generic measures.

Some key limitations of this study need to be mentioned. First, as data collection was carried out by one single interviewer, deep-dives and more detailed understanding of some specific topics might have been influenced by the attitude of the interviewer during the interviews. To tackle this limitation, some actions have been employed such as the interviewer’s self-reflection, peer debriefing, and maintaining an audit trail, that the research team employed to mitigate the potential impact of biases on the study results. We used an existing framework to minimise bias but this may have framed our approach to the analysis. However, cognitive debriefing for face validation with focus on specified aspects of concern in instruments is a standardised approach that is recommended by COSMIN and can therefore help minimise bias. The inclusion of Italian and English speakers in the study, including one researcher involved in the development, also helped to minimise researcher bias. Second, as data were collected during the first wave of the COVID-19 pandemics (i.e., summer of 2020), this might have had an influence on how people felt about their quality of life and overall wellbeing, and the relative importance of some themes. The nature of videoconferencing as method of data collection could have also affected how the participants and the interviewer interacted. These last two limitations have been reported in similar studies conducted in the same time period [22]. Finally, although the study included the relevant target groups who may use the instruments (patients, informal carers, members of the public), it was not possible to consider all the different types of patients or informal carers where these generic instruments could be used due to resource and practical constraints. This includes for example, older individuals in receipt of social care support which is an important group in the Italian context due to the increasing average age of the Italian population. Exploring validity in these groups could be a valuable area for further research, involving a detailed examination of all the items.

Despite these limitations, the current study has also important strengths such as being the first study reporting evidence on content validity of the Italian version of the EQ-HWB and EQ-HWB-S in a sample of Italian patients, informal caregivers and members of the general public, and providing a preliminary proof of the cultural acceptability of these measures.

## Conclusions

The Italian versions of EQ-HWB and EQ-HWB-S showed acceptable face validity in measuring health and wellbeing in an Italian cohort of patients, social care users and informal carers. Some issues with response options were identified, and they might potentially entail the risk of biasing scores obtained. These findings provide useful evidence that can be used to adapt the EQ-HWB and EQ-HWB-S to improve their validity.

## Data Availability

Additional data will be available from Authors upon reasonable request.

## Appendix

**Table 5:** Interviews topic guide

Section	Content
1. Introduction and background questions	• Welcome • Check if the information sheet has been read and participants agree with it • Check if there are any questions on the consent form • Outline of the interview and provide basic information on the topic and the project (i.e., read brief introductory text) • Check permission to record and remind possibility to opt out at any time • Probe for experience with health and/or social care measurement instruments and qualitative questionnaires in general
2. HRQoL and QoL questions	• Probes for meaning of health-related quality of life and quality of life • Probes for important aspects for measuring a health and social care aspects
3. Think-aloud exercise	• Explain think-aloud exercise: “Before we start, I would like to ask you a question in order to practice the think-aloud technique. “Try to visualize the place where you live, think about how many windows this place has. While counting the windows, tell me what you see and what you think.”
4. Explain next steps and Screen-share EQ-HWB questionnaire	• “Please now read the text of the EQ-HWB questionnaire that you have in front of you on the screen and comment aloud on anything that comes to mind as you read the questions. As you read, it is important that you say out loud if you think any of the questions asked contain important aspects of quality of life related to health and health care.”
– Think aloud exercise on EQ-HWB, experimental self-administered version, preceding 1.0 version	
5. Explain ranking exercise	• “I would now ask you to rank five of the questions you have just answered, starting with the question you consider most relevant to measuring aspects of health and social care, and continuing in order to the least relevant, in your opinion.”
– Screen-share EQ-HWB-S questionnaire – Think aloud exercise on EQ-HWB-S, experimental self-administered version, preceding 1.0 version	
6. Verbal probing questions	• Probe for general opinion on the questionnaires • Probe for how easy or difficult it is to answer the questionnaires • Probe for any difficulties in understanding the questions – [If yes] Probe for which questions were difficult to understand and why • Probe for questions that could be removed from the questionnaires • Probe for appropriateness of the short version of the questionnaire
7. Conclusion	• Probe for lack of reference to aspects of health or social care in these questionnaires, that would be considered important • Probe for any other comments to share about anything that was discussed • Thank for the participation.

*Note* The table provided represents the English translation of the original interview topic guide, which was initially formulated in the Italian language

**Table 6:** EQ-HWB and EQ-HWB-S measures^a^

These questions are trying to measure how *your* life has been *over the last 7* *days*. Please answer all questions. There are no wrong or right answers
Difficulty (no, slight, some, a lot and unable) 1. How difficult was it for you to see? (using, for example, glasses or contact lenses if you usually use them) 2. How difficult was it for you to hear? (using, for example, hearing aids if you usually use them) **3. How difficult was it for you to get around inside and outside? (using, for example, walking stick, frame or wheelchair, if you usually use them)** **4. How difficult was it for you to do day-to-day activities? (for example, working, shopping, housework)** 5. How difficult was it for you to wash, toilet, get dressed, eat or care for your appearance?
Frequency (none of the time, only occasionally, sometimes, often, most or all the time) **6. I felt I had no control over my day-to-day life (had the choice or do things or have things done for you as you liked and when you wanted)** 7. I felt unable to cope with my day-to-day life 8. I had trouble remembering **9. I had trouble concentrating/thinking clearly** **10. I felt anxious** 11. I felt frustrated **12. I felt sad or depressed** 13. I felt I had nothing to look forward to **14. I felt lonely** 15. I felt unsupported by people 16. I felt unsafe (fear of falling, abuse or other physical harm) 17. I had problems with my sleep **18. I felt exhausted** 19. I felt accepted by others (felt like you were able to be yourself and that you belonged) 20. I felt good about myself 21. I could do the things I wanted to do
Frequency (items 22, 24: none of the time, only occasionally, sometimes, often, most or all the time) and severity (items 23, 25: no, mild, moderate, severe, very severe) **22. I had physical pain** 23. I had physical pain 24. I had physical discomfort (for example, feeling sick, breathless, itching (not including pain)) 25. I had physical discomfort

*Note* The order of the items reflects an early version of the questionnaire

**Items in bold**: included in the EQ-HWB-S

*ªEuroQol Research Foundation. EQ-HWB*^*TM*^ *is a trade mark of the EuroQol Research Foundation.* Questions in bold are EQ-HWB-S. EQ-HWB-S indicates short version of EQ Health and Wellbeing

**Table 7:** Coding framework for the analysis of response issues

**Comprehension**	
• Odd wording	Participants find the terms or sentence unusual or odd
• Difficult wording	Participants find the terms or sentence difficult because of unfamiliar terms/phrases or struggles with the item’s structure
**Recall**	
• Recall difficulties	Participants find it difficult to recall events to be able to give an answer to the item
**Interpretation**	
• Difficult interpretation of item	Participants express that they do not understand the meaning of an item
• Inconsistency with previous item	Participants’ answer is inconsistent with a previous item
• Wrong interpretation of item	Participants interpret the item in a way that is different than what was intended by the developer of the instrument
• Wide interpretation of the item	Participants focus on more aspects than the ones included in the item by the developer of the instrument
• Narrow interpretation of item	Participants focus on just one aspect of the item or is unsure about the focus of the item
**Response Option Selection**	
• Double-barreled questions	Participants feel that different response options apply to different aspects of the item
• Response options partly applicable	Participants indicate that one part of the response option fits their situation, and one part does not
• Response option is inappropriate	Participants feel that a response option is inappropriate or judgmental
• Irrelevant response options	Participant does not want to answer any of the given response options
• Missing intermediate	Participant feels that there is a gap between two consecutive response options
• Similar response options	Participant feels that two response options are similar
• Disagreement with order of options	Participant does not agree with the order of two response options
• Inconsistent response	Response option chosen did not match what the participants said or the participants’ situation
**Acceptability**	
• Item inappropriate/ judgmental	Participants feel that a question is inappropriate or judgmental and should not be asked
**Relevance**	
• Similar items	Participants could not see the difference between items or feeling that the items are excessively similar
• Item irrelevant	Item not relevant to the participants
• Important QoL asp. missing	Participant feels that the measure misses important aspects of QoL

*Note* Adapted from Penton et al. [24]
